# Molecular response of *Escherichia coli* adhering onto nanoscale topography

**DOI:** 10.1186/1556-276X-7-575

**Published:** 2012-10-18

**Authors:** Loris Rizzello, Antonio Galeone, Giuseppe Vecchio, Virgilio Brunetti, Stefania Sabella, Pier Paolo Pompa

**Affiliations:** 1 Center for Bio-Molecular Nanotechnologies, Instituto Italiano di Tecnologia@UniLe, Via Barsanti, Arnesano, Lecce, 73010, Italy

**Keywords:** Bacteria, Nanotopography, Adhesion, Molecular response

## Abstract

Bacterial adhesion onto abiotic surfaces is an important issue in biology and medicine since understanding the bases of such interaction represents a crucial aspect in the design of safe implant devices with intrinsic antibacterial characteristics. In this framework, we investigated the effects of nanostructured metal substrates on *Escherichia coli* adhesion and adaptation in order to understand the bio-molecular dynamics ruling the interactions at the interface. In particular, we show how highly controlled nanostructured gold substrates impact the bacterial behavior in terms of morphological changes and lead to modifications in the expression profile of several genes, which are crucially involved in the stress response and fimbrial synthesis. These results mainly demonstrate that *E. coli* cells are able to sense even slight changes in surface nanotopography and to actively respond by activating stress-related pathways. At the same time, our findings highlight the possibility of designing nanoengineered substrates able to trigger specific bio-molecular effects, thus opening the perspective of smartly tuning bacterial behavior by biomaterial design.

## Background

Despite the great advancement recently achieved in the development of nanotechnology-based products, as demonstrated by the huge amount of nanomaterials that are present in the market nowadays, a thorough understanding of many biological issues related to these nanotools is still lacking. Among the available nanotech products, nanoengineered biomedical devices are probably one of the most intriguing ones because of their important applications in many research fields, ranging from drug delivery to medical imaging, tissue engineering, and orthopedic implant design [1]. In particular, the fabrication of safe intra-corporeal devices, such as pacemakers, catheters, and bone screws, represents a challenging topic since almost any abiotic surface is prone to contaminations and infections caused by microorganisms that adhere onto the device surface, then colonizing it [2,3]. Bacteria, in fact, mainly live on surfaces rather than as a suspended swimming community, also producing species- and strain-specific extracellular polymeric substance. This may lead to the formation of a complex combination of polysaccharides, external DNA, and catalytic proteins, usually known as biofilms, which is difficult to eradicate and may result in chronic infections [4]. For this reason, many research efforts have been attempted to investigate the physicochemical bases that regulate the bacterium/abiotic substrate interactions. This is a crucial point because hindering the first step of the adhesion event likely represents the only opportunity to block further biofilm growth and development [3]. In particular, a wide range of substrates presenting different surface chemistries, physical characteristics, and surface topographies has been designed and investigated to date in order to understand which physicochemical cue can avoid bacterial adhesion and persistence [5-9]. In this respect, particular attention has been focused toward the effects of surface micro- and nanostructuration over bacterial attachment, obtaining, however, rather contrasting results. Using multiple linear regression analysis, Bakker et al*.* showed, for instance, a direct relationship between surface roughness and the number of adherent bacteria on polyurethane-coated glass plates [10]. The importance of the size and morphology of nanoscale features has also been addressed by other works, which confirmed the trend reported by Bakker, showing a general increase in the number of adherent bacteria with increasing surface nanoroughness [11,12]. On the other side, other studies found out an opposite trend, namely that a decrease in the topographical feature size leads to an increase in the number of attached bacteria [13]. In this respect, many of these studies mainly focused the attention on the theoretical and physicochemical point of view in studying the interaction between abiotic surfaces and bacteria. It should be, however, considered that, since microorganisms are rapidly evolving living systems, they are also able to sense and actively respond to surface cues. Bacteria have, in fact, fine molecular and mechanochemical sensors as well as highly controlled intracellular signaling pathways whose changes in activities with respect to surface nanotopography-related stimuli are nearly completely unknown so far.

In this work, we aimed to investigate the molecular mechanisms underlying the early stage of bacterium/abiotic substrate interaction at the interface. After detecting some important changes in their morphological features, we explored the expression level of several genes in *Escherichia coli* cells adhering onto flat and nanostructured gold surfaces, detecting the activation of the two-component system stress pathways CpxP/R and the up-regulation of the fimbrial recombinase FimE. These results suggest that nanostructured gold surfaces lead to a general stress condition in adherent bacteria, which down-express and degrade their adhesive organelle type-1 fimbriae and activate their recovery pathway to remove misfolded periplasmatic proteins. These findings highlight how surface nanotopography may play a pivotal role in triggering and guiding specific biological outcomes.

## Methods

### Substrate fabrication and characterization

For substrate preparation, we exploited a method already discussed elsewhere [14,15]. Briefly, NH_2_-modified glass slides were coated with 50 nm of Au film by thermal evaporation (0.8 Ǻ/s) in order to obtain a very flat and uniform gold film. Nanorough Au films were achieved by coating 50 nm of Ag film (1.5 Ǻ/s) onto gold pre-coated glass substrates first and then immersing them within a solution of 10^−3^ M HAuCl_4_ for 15 min. The surface topography of flat and nanostructured substrates was investigated by scanning electron microscopy (SEM; Nova NanoSEM200, FEI, Hillsboro, OR, USA). Samples were positioned at a working distance of 5 mm and scanned with an 18-KeV e-beam. The substrate line profile was inspected by atomic force microscopy (contact mode in air) using the commercial nanoscope IVMultiMode SPM (Veeco Instruments, Santa Barbara, CA, USA) under ambient conditions (20°C to 25°C, atmospheric pressure, approximately 50% humidity).

### Bacterial strain and growth conditions

A loop of glycerol stock of *E. coli* strain TG1 (K12, *lac-pro supE thi hsdD5* (F′*traD36 proA*^*+*^*B*^*+*^*lacI*^*q*^*lacZ* M15)) was streaked onto a Luria-Bertani medium agar plate and incubated overnight at 37°C. Then, a single colony was picked and grown in Luria-Bertani (LB) liquid medium overnight at 37°C up to an optical density at 600 nm (OD_600_) of 1.00 ± 0.05 (corresponding to *c.a.* 8 × 10^8^ cells/mL) in a shaking incubator (240 rpm). The overnight culture was diluted in LB medium to an OD_600_ of 0.1 and transferred into a six-well plate containing the substrates. The plates were incubated at 37°C for 12 h with shaking (240 rpm). After the incubation, the surfaces were gently rinsed four times with 0.2 M Tris, pH 7.5 to analyze only surface-associated bacteria.

### Confocal microscopy analyses

To count the number of adherent bacteria, substrates were immersed in 4% formaldehyde (to fix cells) and then stained with Hoechst 33258 (1 μg/mL final concentration); imaging was performed using a confocal microscopy (Leica TCS-SP5 AOBS, Solms, Germany), and direct counting was carried out on flat and all the nanorough samples. For each replicate (three independent replicates were used), eight scan fields of 400×400 μm^2^ were analyzed.

### Real-time quantitative PCR

The expression of ten different genes (namely *luxS*, *ompC*, *lpxC*, *murA*, *dsbA*, *fliC*, *cpxR*, *cpxP*, *degP*, and *fimE*) was investigated for bacteria grown on flat and nanorough gold substrates, for 12 h, by real-time quantitative polymerase chain reaction (qPCR). The *gapA* gene, encoding d-glyceraldehyde-3-phosphate dehydrogenase A, was used as an independent internal control. After the incubation with substrates (three independent biological replicates), the surfaces were gently rinsed four times with 0.2 M Tris, pH 7.5 to analyze only surface-associated bacteria. The total RNA was extracted from bacterial cells of each sample (namely flat and nanorough gold substrates) using TRI Reagent (Sigma-Aldrich, St. Louis, MO, USA), as described in the manufacturer's instructions, giving special attention to detach only adherent bacteria. The amount of mRNA of each sample (flat and rough gold) was determined by taking the optical density 260:280 ratio using a UV–vis spectrophotometer, and RNA quality was analyzed using agarose gel electrophoresis (1.2%, 70 V for 30 min; data not shown). First-strand cDNA was prepared from 2 μg of total RNA using enhanced avian reverse transcriptase (Sigma-Aldrich) and random nonamer (Sigma-Aldrich) primers in 20 μL reaction volume, and 2.5 μg was digested with RNase (Sigma-Aldrich). The real-time (RT)-qPCR was carried out using the primer sequence reported in Table 1. PCR was performed with an ABI 7500 thermal cycler (Applied Biosystem, Carlsbad, CA, USA) following the manufacturer’s suggestions, using SYBR Green-based detection of PCR products. Melting curves were examined after amplification to confirm single product measurement. For each gene, we used 10 ng of cDNA mixed with 10 μL of 10X Express SYBR Green qPCR SuperMix premixed with ROX (Invitrogen, Grand Island, NY, USA), 2 μL of 4 μM gene specific primers mix, and 7 μL of DEPC-treated water. Reaction conditions for all genes were as follows: initial denaturation at 95°C for 10 min followed by 40 cycles of 15 s at 95°C and 1 min at 60°C. This program was followed by a melting curve program (60°C to 95°C with a heating rate of 0.1°C/s and continuous fluorescence measurements). Relative expression was calculated from cycle threshold values (ΔΔCt method) using *gapA* gene, encoding d-glyceraldehyde-3-phosphate dehydrogenase A, as an independent internal control. The primers used in real-time qPCR analyses were designed by the on-line Primer-BLAST software of NCBI, whose list is reported in Table 1.

**Table 1:** Primers used in real-time qPCR analyses

**Gene name**	**Primer forward**	**Codified protein**	**GenInfo identifier**
	**Primer reverse**		
*gapA*	TTGGCCGTATCGGTCGCATT	Glyceraldehyde-3-phosphate dehydrogenase A	16129733
	AAACGGCCGTGAGTGGAGTC		
*ompC*	TAACGGCGACCGTGCTGAAA	Outer membrane porinprotein C	16130152
	ACGCGAGTTGCGTTGTAGGT		
*luxS*	ACCGTGTTCGATCTGCGCTT	S-ribosylhomocysteinelyase	16130599
	GCAAACAGGTGCTCCAGGGT		
*murA*	AACGAAGCTCCAGGGCGAAG	UDP-N-acetylglucosamine 1-carboxyvinyltransferase	16131079
	TTCGCACCCAGCTGGCTTAG		
*fliC*	CAGTTCTCCAACCGCGGTCA	Flagellar filament structural protein (flagellin)	16129870
	GAGCCTCACCACCAGCAGTC		
*lpxC*	GCAACCAGCGCTATGCGATG	UDP-3-O-acyl N-acetylglucosaminedeacetylase	16128089
	AGGCACAAACCACGGGACTG		
*slp*	CCGACTGCTCGCCAGACAAA	Outer membrane lipoprotein	90111603
	CACACCTGGATGCCCTGCAT		
*flu*	TGCCGGCACGGTCCGGGATGA	CP4-44 prophage; antigen 43 (Ag43) phase-variable biofilm formation autotransporter	49176177
	GCCCCGGGCGCGGAAGTCGT		
*cpxP*	GGCCCGGCACGAACAGCCTCCT	Periplasmic adaptor protein	49176443
	ACCGCTTGCTGCTCCGGCGT		
*dsbA*	CACAAGGCCGGGGCGCGTGG	Oxidoreductase that catalyzes reoxidation of DsbA protein disulfide isomerase I	49176085
	AGCGCAGCACCCAGAACGCCGA		
*degP*	GCGCTGGGGCGTAGCGGCCT	Serine endoprotease (protease Do), membrane-associated	16128154
	TGCCGCCGTCCGGTGCGAGG		
*cpxR*	TCTGGCTGACGCTGGCGCTGGT	Sensory histidine kinase/signal sensing protein	16129840
	CGCCCGGAACAGACGCCGCCA		
*fimE*	GTTACGGGGCAACGGGAGCC	Tyrosine recombinase/inversion of on/off regulator of fimA	16132134
	CTGGGTCCAGCGTTCCACGG		

## Results and discussion

**Figure 1:**
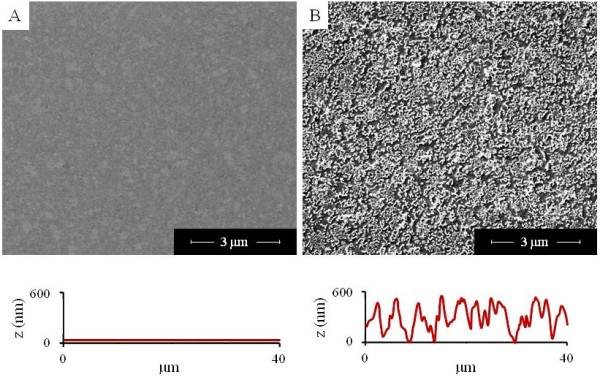
**Substrate characterizations.** SEM investigation of flat **(A)** and nanorough **(B)** gold substrates, with their respective AFM line profiles at the bottom part of the pictures.

We investigated the molecular basis of interaction between *E. coli* cells and metal substrates presenting different surface nanotopographies (namely, flat and nanorough gold). For substrates fabrication, we exploited a method discussed elsewhere [16,17]. Briefly, we used a spontaneous galvanic replacement reaction (SGDR), which allows metal deposition in the absence of an external reducing agent [18,19]. This electroless plating approach is fairly cheap, highly reproducible, and enables the fabrication of metal films with highly controlled surface topographies that are uniform over wide areas. Representative SEM images of flat and nanorough gold substrates are reported in Figure 1. In particular, as shown in Figure 1A, a homogeneous, flat gold film was used as reference substrate. On the other side, the rough Au film (Figure 1B) obtained by SGDR shows a randomly organized and uniform nanoroughness, presenting hollow/porous nanostructures that are regularly extended over a wide area. We also carried out atomic force microscopy (AFM) characterizations of the substrates. In particular, the AFM line profiles (Figure 1, bottom) illustrate that, while flat gold surfaces display a clear smooth profile (with a *R*_a_< 1 nm), the nanostructured Au surfaces have an average roughness profile of *c.a.* 100 nm. These substrates were exploited to investigate the early stage of *E. coli* adhesion capability, focusing on the possible activation of specific bio-molecular pathways.

**Figure 2:**
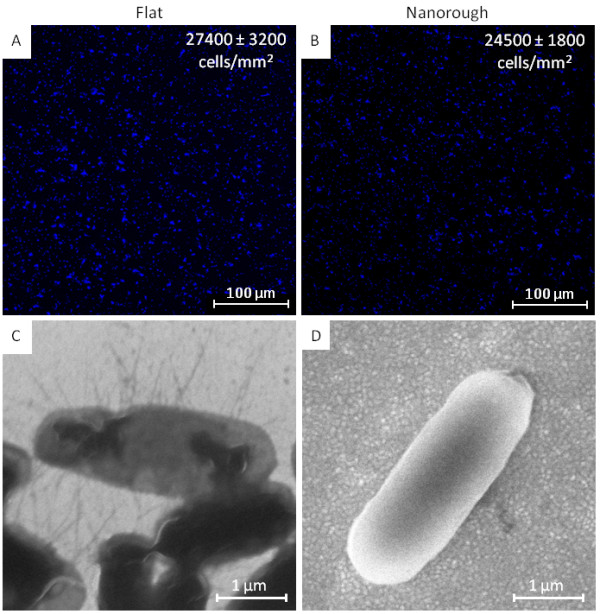
**Impact of nanoroughness on the morphology of *E. coli*.** Confocal microscopy images of DAPI-stained bacteria growing onto flat **(A)** and nanorough **(B)** gold substrates revealing that the total number of bacteria is almost the same between the two samples. SEM pictures of *E. coli* cells growing onto flat **(C)** and nanorough **(D)** gold substrates showing the loss of type-1 fimbriae expression.

We first performed a preliminary counting experiment of bacteria growing onto the two distinct substrates in order to verify whether nanotopography may affect the number of adhering bacteria. Then, we carried out morphological investigations, by AFM, to detect any phenotypical changes of microorganisms upon interaction with the different nanotopographies. Experimental data show that, although surface nanoroughness does not directly influence the adhesion capability of *E. coli* cells in terms of total number of adherent cells (Figure 2A,B), it significantly impacts their adaptation, morphology, and physiology (Figure 2C,D). In particular, the bacterial colonization on the abiotic surfaces was not found to rely on nanoscale changes in surface nanoroughness as the average density of adherent bacteria was practically the same in the two samples (Figure 2A,B). It should be mentioned that, in this case, the two substrates display similar wettability properties, so such surface parameter does not play a significant role in the bacterium/substrate interaction. In fact, albeit nanorough gold surfaces are fairly hydrophilic (with a static water contact angle(WCA) of approximately 25°) as compared to their flat counterpart (static WCA of approximately 85°), after incubation with the bacterial culture medium, both nanotopographies acquire a rather hydrophilic character (WCA of approximately 10° and 30° for nanorough and flat samples, respectively). This is due to the adsorption of medium proteins onto the gold surfaces, which leads to a variation in the wettability properties of the substrates toward hydrophilicity, regardless of the original surface properties [14]. Consistent with the literature, our results confirm the contrasting data regarding the influence of surface nanotopography and wettability on bacterial adhesion (e.g., the reported increase or decrease in the number of adherent bacteria as a function of surface nanoroughness). This suggests that a general explanation or theory about the adhesion mechanism is not feasible since bacterial interaction and persistence on abiotic surfaces are strongly dependent on the specific physicochemical properties of the substrates employed as well as on the bacterial strains used (e.g., Gram-positive or Gram-negative) and their growth conditions (i.e., incubation time, growth medium, ionic strength of the medium, temperature, shaking/flowing or static incubations).

**Figure 3:**
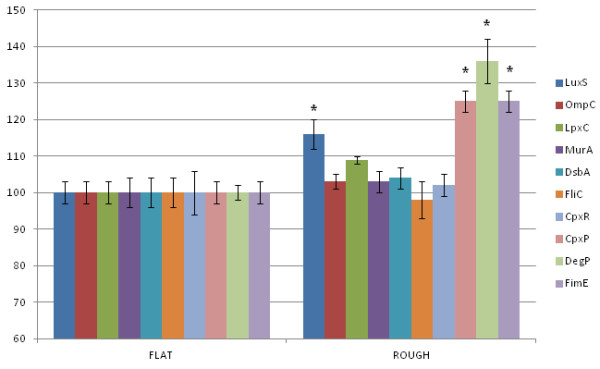
**Results of RT-qPCR.** RNA expression level (%), by RT-qPCR, of *E. coli* growing onto flat (left histograms) and nanorough (right histograms) substrates. All data relative to PCR experiments were analyzed by a statistical software to evaluate the significant difference with respect to the control (a star indicates *p* value = 0.001).

Notably, Figure 2C,D shows that the population of *E. coli* adhering onto nanostructured surfaces underwent an important phenotypical change with respect to those adhering onto flat films. Specifically, the SEM investigations illustrate that *E. coli* growing onto flat gold film strongly adhered onto the surface, as demonstrated by the presence of the type-1 fimbriae. Such structures are, in fact, adhesive organelles that bacteria employ to contact and robustly interact with both host cells and abiotic surfaces [20,21]. They also promote biofilm formation and development [22,23]. On the contrary, bacteria attached onto nanostructured surfaces did not phenotypically display type-1 fimbriae, thus suggesting a weak interaction with the surfaces. This latter finding highlights that, although the total number of adherent bacteria is roughly the same, *E. coli* cells growing onto nanostructured substrates exhibit the typical features of cells that are not able to make a correct and strong interaction with the surface. In a previous study, we found out that nanotopography may induce important changes in fimbrial expression, mainly related to the over-expression of one fimbrial operon repressor, namely LrhA; the detailed molecular activity of LrhA, however, has not been completely clarified yet [14]. In this work, we aimed at uncovering the molecular mechanisms underlying fimbrial expression as a function of surface-related physical stimuli as well as to understand the molecular bases of bacterium/abiotic substrate interaction at the interface in the early stage of adhesion event. In particular, we incubated *E. coli* with the two different nanotopographies and investigated the expression level of several genes that are involved in fimbrial synthesis, inter- and intra-species communication, biofilm formation, response to stress stimuli, and adhesion to both host cells and abiotic surfaces. The results of RT-qPCR of bacteria growing onto nanorough surfaces, compared to the reference flat substrate, are illustrated in Figure 3.

Notably, we found a significant over-expression of *cpxP* and *degP* genes, which are involved in the bacterial envelope stress response, named as Cpx two-component system [23]. This pathway is activated by the presence of large amounts of misfolded fimbrial protein aggregates, which are associated with the inner membrane. In particular, the periplasmic fimbrial misfolded subunits titrate *cpxP* and further activate *cpxA*; the latter then shifts its own phosphatase activity to a kinase and autokinase activity, leading to an accumulation of a phosphorylated transcription factor CpxR in the cytoplasm. This protein activates the expression of envelope folding and degrading factors, including *dsbA* and *degP*. However, as indicated by our data, bacteria growing onto nanorough Au surface do not up-regulate the periplasmic protein disulfide isomerase *dsbA*, which is involved in protein quality control and refolding processes. On the other side, the over-expression of *degP* suggests that *E. coli* cells prefer to shift their molecular activity on removing misfolded proteins in the periplasmic space by degrading them, instead of trying to refold them, most probably because of the high presence of extremely unfolded/damaged proteins. Moreover, we found that bacteria growing onto nanostructured gold substrates over-express the *fimE* gene. *fimE* encodes for a recombinase protein involved in the on-to-off fimbrial switching (i.e., FimE), leading bacteria to repress the type-1 fimbrial synthesis under particular conditions [24,25]. These data are in good agreement with the SEM investigation of Figure 2 and better explain also our previous findings [14].

**Figure 4:**
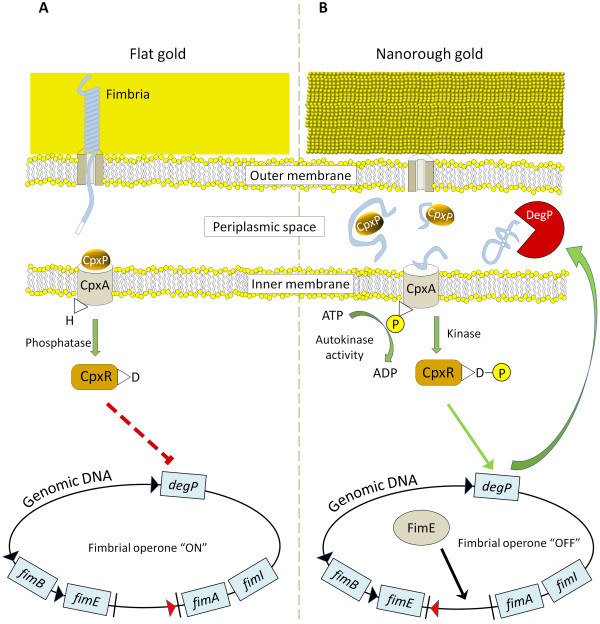
**Scheme of the molecular mechanism of bacteria/nanorough substrate interaction. (A) ***E. coli* growing on flat gold substrates present the typical type-1 fimbriae; the two-component system Cpx pathway is inactive, thus CpxA posses a phosphatase activity which inhibits the transcription factor CpxR, resulting in a repression of *degP* transcription. At the same time, the promoter of the fimbrial operon is in the ON orientation. **(B)** The nanorough gold substrates lead to a general stress condition in *E. coli*, inducing a high presence of unfolded/misfolded proteins in the periplasmic space. Such misfolded proteins titrate CpxP from CpxA, whose activity shifts from phosphatase to autokinase and kinase, thus activating CpxR. This protein activates the transcription of *degP* which degrades unfolded proteins of the periplasmic space. Moreover, FimE switches the operon of the fimbrial promoter in the OFF orientation.

Taking into account all these data, it is likely that *E. coli* adhering onto nanostructured gold substrates undergo a general stress condition, which results in two distinct biological responses: (1) The two-component system Cpx pathway ‘senses’ the external stimulus (i.e., the nanoscale variation of surface roughness) by detecting periplasmic and/or external misfolded proteins (thanks to the *cpxP* recruitment), including the fimbrial subunits; as a consequence, bacteria activate the *degP*-related degradation of fimbrial proteins for the recycle of amino acids. (2) FimE recombinase is over-expressed, which switches off the fimbrial operon, thus inhibiting the transcription of all the fimbrial subunits. As a result, bacteria adhering onto nanorough gold substrates repress the fimbrial transcription and, at the same time, degrade the fimbrial protein subunits, which are present in the periplasmic space. The scheme in Figure 4 summarizes the possible molecular mechanisms involved in the bacterium/nanotopography interaction. This is also consistent with our previous proteomic data, in which some proteins involved in general stress response were found to be up-regulated in *E. coli* attached onto rough substrates [14].

We also found an up-regulation of *luxS* gene in the nanorough samples. Such gene is involved in the biosynthesis of a quorum sensing (QS) autoinducer molecule (AI-2), which has been demonstrated as a universal signal that could be used by a variety of bacteria for communication, also among different species [26]. QS molecules are used by microorganisms to coordinate the gene expression also of the surrounding community, thus enabling bacteria to behave like a *quasi* complex multicellular organism. This phenomenon occurs when bacteria have to overcome some environmental difficulties; in our case, such stress condition is represented by the nanotextured substrates.

On the other hand, the *ompC* gene, which codifies for the outer membrane porin C, *lpxC*, which is required for lipid A expression, and *murA*, which is important for external wall synthesis, are not regulated upon interaction with the nanostructured substrates. Also, the *fliC* gene that codifies for a flagella subunit, as well as *cpxR*, which is an effector of the two-component system CpxR-A pathway, is not regulated in the treated samples. In this respect, we can envisage that, although nanostructured Au substrates strongly impact the bacterial adhesion capability, the genes codifying for the biofilm expression [27] seem to be unregulated in the early stage of the adhesion event. Further and more systematic studies are required in order to evaluate any possible influence of nanotopographies on biofilm formation after longer incubation periods. On the other hand, our data suggest that the mechanosensing machinery of *E. coli* feels the change in surface nanotopography as a physical stress signal. Hence, the bacteria focus their molecular activities on regulating and triggering specific pathways, which are important for recovery from stress conditions.

## Conclusions

A detailed understanding of the molecular mechanisms underlying the interactions between nanomaterials and living systems is fundamental for providing more effective products for nanomedicine and drug delivery. The ability to smartly control the response of bacteria by tuning specific physicochemical properties of the nanosurfaces is ultimately the challenging goal. However, in studying nano-biointeractions, it is imperative to take into account the dynamic evolutions of the biosystem/abiotic substrate interaction events. In this context, we have demonstrated that nanostructured gold substrates induce significant changes in the morphological and genetic response of adherent *E. coli*. Particularly, we found out that nanotopography induces the activation of the stress signaling two-component system Cpx pathways and up-regulation of the fimbrial recombinase FimE. This data suggest that bacteria possess an extra-fine mechanosensing machinery, which is able to detect even nanoscale features in abiotic surface nanotopographies. Finally, this work may pave the way to the design of a new generation of devices which are able to trigger and tune specific biological outcomes.

## Competing interests

The authors declare that they have no competing interests.

## Authors’ contributions

LR fabricated and characterized the substrates, carried out the bacterial culture, selected the genes to be investigated, and drafted the manuscript. AG and GV carried out the molecular genetic studies and performed the statistical analyses. VB carried out the confocal investigations. SS participated in the design of the study. PPP conceived of the study, participated in its design and coordination, and drafted the manuscript. All authors read and approved the final manuscript.
